# Chemistry Routes for Copolymer Synthesis Containing PEG for Targeting, Imaging, and Drug Delivery Purposes

**DOI:** 10.3390/pharmaceutics11070327

**Published:** 2019-07-11

**Authors:** Kamil Rahme, Nazih Dagher

**Affiliations:** Department of Sciences, Faculty of Natural and Applied Sciences, Notre Dame University-Louaize, Zouk Mosbeh, P.O. Box 72, Zouk Mikael, Lebanon

**Keywords:** polyethylene glycol (PEG), PEG-conjugates, nanocarriers, stabilization, stealth character

## Abstract

Polyethylene glycol (PEG) is one of the most frequently used polymers for coating nanocarriers to enhance their biocompatibility, hydrophilicity, stability, and biodegradability. PEG is now considered to be among the best biocompatible polymers. It offers sterical hindrance against other nanoparticles and blood components such as opsonin, preventing their macrophage phagocytosis and resulting in a prolonged circulation time in blood stream, consequently a ‘stealth character’ in vivo. Therefore, PEG has a very promising future for the development of current therapeutics and biomedical applications. Moreover, the vast number of molecules that PEG can conjugate with might enhance its ability to have an optimistic perspective for the future. This review will present an update on the chemistry used in the modern conjugation methods for a variety of PEG conjugates, such methods include, but are not limited to, the synthesis of targeting PEG conjugates (i.e., Peptides, Folate, Biotin, Mannose etc.), imaging PEG conjugates (i.e., Coumarin, Near Infrared dyes etc.) and delivery PEG conjugates (i.e., doxorubicin, paclitaxel, and other hydrophobic low molecular weight drugs). Furthermore, the type of nanoparticles carrying those conjugates, along with their biomedical uses, will be briefly discussed.

## 1. Introduction

Bioconjugation is a technique used by chemists to bond two molecules in which one of them is a biomolecule. When the other molecule is Poly (etheylene glycol) (PEG), another term arises which is PEGylation. Poly (ethylene glycol) is known to be a neutral polymer and is now one of the most popular polymeric materials used for alteration and control of bio distribution. Moreover, pharmacokinetics and often toxicity of bio active molecules can be affected strongly by PEGylation, for example Cassettari et al. demonstrated that mPEG-*g*-chitosan conjugates exhibited reduced toxicity toward cells, as compared to unmodified chitosan counterparts [1]. Moreover, PEG may increase the lifetime of the “drug-carrier” assembly, thus resulting in the administration of lower concentrations of the “drug-carrier” composite and consequently lowering toxicity [2,3]. PEG holds a wide range of advantageous properties that include high solubility in aqueous media as well as organic solvents making it easy for end-group modification [4]. It is also widely used for modification of carriers used in therapeutics [5] because PEG offers a shielding character that avoids rapid renal excretion from the body. This shielding property is also called the “stealth character” effect that PEG offers through its reduced interaction with blood components mainly “opsonin” that is well known to enhance phagocytosis (opsonization) and subsequently inhibiting its uptake by the reticuloendothelial system (RES) [6,7]. Moreover, nanoparticles are well known to be sensitive to high ionic strength media and may aggregate in buffer and complex biological media due mainly to increased van der Walls attraction (protein etc.) [8]. Coating nanoparticles with a neutral PEG layer can stabilize nanoparticles in such complexes and high ionic strength media. The resulting PEGylated nanoparticles have fewer tendencies to aggregate due to the “conformational cloud” causing steric stabilization. This “conformational cloud” is responsible for the reduced interactions with the blood and tissue components resulting in PEGylated macromolecules offering less immunogenicity and antigenicity [9,10]. Although PEG is considered to be a non-bio-degradable macromolecule, researchers have demonstrated that it can be easily excreted from the body by the kidneys [11,12,13,14].

During the 1930s, PEG was synthesized commercially by the base initiation of the addition of ethylene oxide to ethylene glycol and diethylene glycol [15], and it is now commercialized with different molecular weights and functionalities. Low molecular weight molecules such as oligonucleotides, siRNA, and low molecular weight drugs are usually PEGylated with 20–50 KDa PEG, while the incorporation of larger molecules such as antibodies or particulate systems require PEG with 1–5 KDa molecular weight. PEG of about 3–4 KDa is usually administered as a laxative i.e., GoLYTELY and MoviPrep [9].

**Figure 1:**
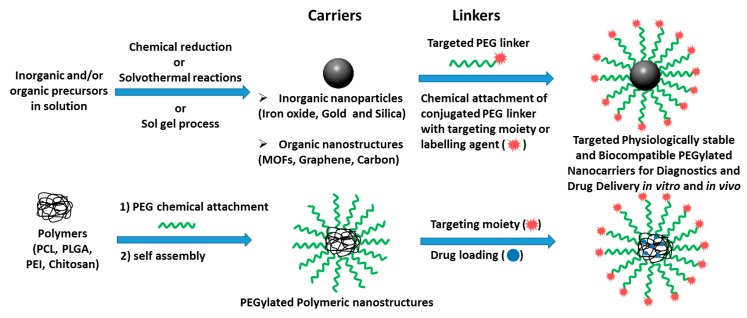
Nano particulate delivery designs discussed in this review.

As a result of the above, Poly (ethylene glycol) modified nanocarrier systems can act accordingly to any specific purpose (from targeting to delivery). This review will discuss Bio-conjugated PEG Based Polymers Modified Nanocarriers acting as targeting vehicles, imaging tools, and drug carriers. Furthermore, nanocarriers come in many different designs. Two general setups are shown in the illustration below. However, in this review the conjugation, modification, and brief medical uses of the nano particulate designs only shown in Figure 1 will mainly be discussed.

A list of the different types of nanoparticles used as carriers discussed in this review, as well as the pathways followed for their synthesis are also summarized in Table 1.

## 2. Synthesis of Modified Bio-Conjugated PEG Nanoparticles Acting as Targeting Agents

Some pathogenic sites do not allow or obstruct enhanced permeability and retention (EPR), which presents a challenge in delivering macromolecules to those sites [28]. One way for overcoming this situation is by functionalizing nanoparticles with certain ligands that can selectively target and bind to those surface receptors of those pathogenic cells. Among the most used targeting ligands are usually small molecules. Small molecules are known for their stability under physiological conditions, as well as their easy conjugation with coated nanoparticles specifically PEGylated ones, and having a low cost, considering its synthesis in high yield [29].

### 2.1. Poly(ε-caprolactone)-block-Poly(ethylene glycol)-Biotin (PCL-PEG-Biotin) Synthesis and Biomedical Application Overview

**Figure 2:**
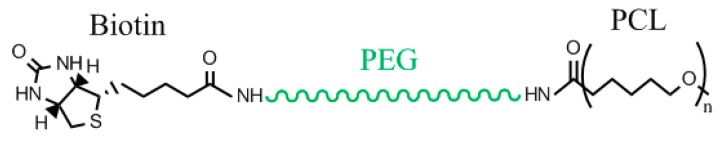
Chemical connectivity of PCL-PEG-Biotin nanoparticle design.

Biotin-conjugated PEG-PCL Block Copolymers performing as targeting nanocarriers can be prepared starting with the activation of Biotin. *N*,*N*′-Dicyclohexylcarbodiimide (DCC) is used as the cross linker. DCC activates the Biotin at its carboxylic group end to make the oxygen a better leaving group and the primary amine of bi-functional PEG-amine substitutes for the activated Biotin-DCC intermediate [16,30]. The product biotin-conjugated PEG is then copolymerized with ε-caprolactone. The other amine group of the biotin-conjugated PEG acts as the initiator for the reaction. The reaction is driven by having an electron deficit carbonyl carbon on the monomer ε-caprolactone due to the withdrawing ability of the oxygen atom, favoring the ring opening of ε-caprolactone in the presence of a nucleophile. ε-caprolactone is attacked by the nucleophilic nitrogen atom of the amine in this case. The new formed species is now considered to be the nucleophile because the alkoxide ion of the opened ε-caprolactone has a stronger nucleophilic character than a partial negatively polarized nitrogen atom [31]. The desired composition of the polymer shown in Figure 2 could be altered by changing the feed ratio of ε-caprolactone monomer.

A macromolecule vehicle can now be constructed having a micelle structure. Usually chemists make use of the micellar structure and entrap a hydrophobic drug inside. An example is the incorporation of paclitaxel, an anticancer drug, to form Biotin-PEG-PCL paclitaxel loaded nanomicelles with sustained release of the drug. Biotin-PEG-PCL paclitaxel loaded nanomicelles were synthesized to be used in targeted chemotherapy for cancer. It was shown that micelles with targeting ability incorporating the Biotin biomolecule showed a higher drug uptake in MCF-7 and HeLa cells against free paclitaxel formulation, conferring better cell targeting and a much higher cytotoxicity for cancer cells expressing a Biotin receptor [16]. In another example, pillararene-based amphiphilic supramolecular diblock polymer (P5-PEGBiotin⊃PCL-C2V) based on the host–guest recognition between a water soluble pillar[5]arene dimer, and a viologen salt self-assembles into polymersomes which were used as smart nanocarrier vehicles to deliver the anticancer drug doxorubicin hydrochloride (DOX) preferentially to biotin receptor over-expressing cancer cells (HeLa cells). Moreover, in vivo studies revealed that the DOX-loaded PEGylated supramolecular polymersomes could prolong the circulation time in the bloodstream and conserve the antitumor efficacy [32]. In another study, Nosrati et al. synthesized Biotin-PEG-PCL by ring polymerization method of anhydrous caprolactone monomer to a biotin-PEG previously conjugated by DCC/NHS coupling as the reaction initiator and tin(II) octoate catalyst. The authors demonstrated that Biotin-PEG-PCL nanomicelles could be loaded with artemisinin (ART) that was slowly released in a pH controlled manner, resulting in an inhibitory effect on MCF-7 breast cancer cells and no toxic effects on HFF2 cells. Moreover, anticancer activity in vivo showed an increase in the accumulation of substances in the tumors leading to a decreased volume of the tumor in the 4T1 breast cancer model [33].

### 2.2. Magnetite-PEG-Folate Synthesis and Biomedical Application Overview

To start the synthesis of Folate conjugated PEG nanocarriers, magnetite (Fe_3_O_4_) nanoparticles (MNPs) can be synthesized by a convenient and cheap method that is the co-precipitation of the two salts FeCl_3_·6H_2_O and FeCl_2_·6H_2_O [34]. The magnetite is usually coated to protect the nanoparticles from oxidation and provide stability against agglomeration.

To achieve this task, the magnetite nanoparticles can be coated with organic molecules, i.e., small organic molecules or polymers, also coating with an inorganic layer, such as silica, metal, metal oxide, or metal sulfide. Logically, it is convenient that the protecting layer does not only stabilize the NPs but can also be used for further functionalization. PEG polymer would be a suitable choice. However, before PEGylation, during synthesis oleic acid and oleylamine are added to stabilize the formed magnetite nanoparticles. Both the carboxyl (-COOH) group and amino (-NH_2_) group attach to the magnetite nanoparticles through coordinate bonds [35]. 

**Figure 3:**
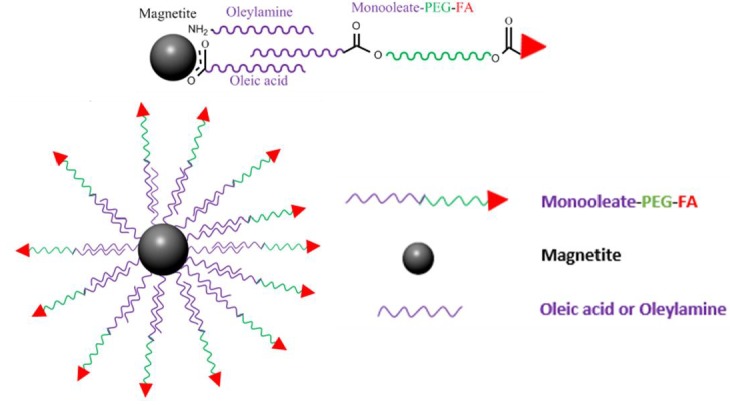
Magnetite-PEG-Folate nanoparticle, explaining how Folic acid (FA)-PEG-monooleate can form interdigitated bilayers to transfer hydrophobic magnetite into aqueous media, resulting in a biocompatible nanoparticulate system.

PEG monooleate can now be added to the magnetite nanoparticles and sonicated. The PEG is now chemisorbed with iron oxide nanoparticles by hydrophobic-hydrophobic interactions between the oleate part of the PEG monooleate and oleyl on the magnetite [17]. The resulting product is now MNPs-PEG and it is added to previously DCC activated Folic acid (FA). The final product is MNPs-PEG-Folate, shown in Figure 3. This macromolecule can now be used to target cancer cells overexpressing the folate receptor. Doxorubicin loaded FA-PEG-MNPS were able to efficiently kill HeLa cells. FA-PEG-MNPs themselves were non cytotoxic, therefore FA-PEG-MNPS could have the potential to be a successful drug delivery system in cancer chemotherapy [17]. Yoo et al. synthesized PEG-Folate via DCC/NHS coupling; the latter was grafted onto aminosilane-immobilized superparamagnetic iron oxide nanoparticles (SPIONs) via the NHS/DCC coupling method. Moreover, cysteine was also grafted on the FA–PEG–SPIONs and the resulting FA–PEG–SPIONs–Cy5.5 showed receptor mediated endocytosis into KB cells and lung cancer model mice as confirmed from confocal microscopy and fluorescent flow cytometry [36]. Li et al. synthesized alendronate ALN-PEG-FA and PCL-PEG-FA coated magnetite nanoparticles and studied their in vivo biodistribution after systemic administration into mice by magnetic resonance imaging magnetic resonance imaging (MRI) [37]. In a recent study, Rajkumar and Prabaharan reported on the synthesis of multi-functional nanocarriers based on iron oxide nanoparticles conjugated with doxorubicin (DOX), poly (ethylene glycol) (PEG), and folic acid (FA) (IO-MMA-DOX-PEG-OCH3/FA) using mono-methyl adipate (MMA) as a linker, and an acid-cleavable (pH 5.6) hydrazone bond to attach DOX. The IO-MMA-DOX-PEG-OCH3/FA nanoparticles were used to deliver DOX to HeLa cells via folate-receptor-mediated endocytosis, and demonstrated enhanced cytotoxicity against HeLa cells through apoptosis [38].

### 2.3. Poly(lactic-co-glycolic acid)-Block-Poly(ethylene glycol)-Mannose (PLGA-PEG-Mannose) Synthesis and Biomedical Application Overview

**Figure 4:**
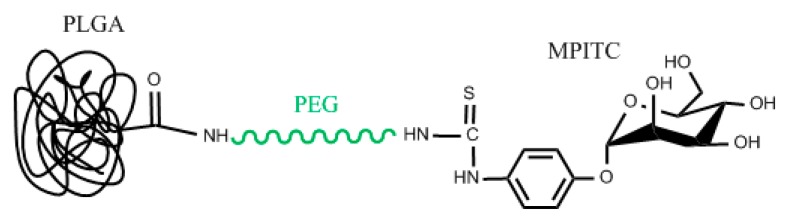
Chemical connectivity of PLGA-PEG-Mannose nanoparticle design.

This macromolecule can be readily synthesized by starting with the PEGylation of mannose by PEG bis-amine derivative. α-d-mannopyranosylphenyl isothiocyanate (MPITC) has an isothiocyanate functional group. Isothiocyanates are weak electrophiles, akin to nucleophilic attack at the carbon of the –N=C=S group [39]. In the case of PLGA-PEG-Mannose shown in Figure 4, the primary amine of the PEG bis(amine) acts as the nucleophile. Mono t-BOC-protected PEG bis(amine) is added to a MPITC solution, and with the right conditions, PEG-mannose is produced [40,41]. Following PEG-mannose synthesis it can be then connected to a carrier. Among several carriers that can be used, PLGA will be discussed here. After removing the t-BOC group to activate the other amine of the PEG, amine-PEG-mannose can now continue reacting. Previously activated PLGA using DCC and NHS to get an intermediate PLGA-NHS product that is capable of forming an amide bond with the amine of NH_2_-PEG-mannose is reacted with NH_2_-PEG-Mannose. The final structure is PLGA-PEG-Mannose [18]. PLGA-PEG-Mannose nanoparticles can now be prepared using an emulsion solvent evaporation (oil in water (o/w) emulsification) technique [42]. During this step, a suitable drug against macrophages such as amphotericin B can be dissolved to get entrapped in the PLGA.

The engineered nanoparticles with mannose as a targeting agent show successive targeting ligand ability. Moreover, those engineered Poly(lactic-co-glycolic acid) nanoparticles (PLGA NPs) with a PEG spacer compared to those where mannose is directly attached to PLGA, indicated enhanced uptake, potential antileishmanial activity, and greater disposition in macrophage-rich organs, signifying improved macrophage targeting [18]. Zhu et al. reported an acid-sensitive PEG shedding mannose-modified nanoparticle platform that targets tumor-associated macrophages (TAMs) specifically via mannose-mannose receptor recognition present on the surface of macrophages, and their results demonstrated that uptake by normal macrophages in the mononuclear phagocyte system (MPS) organs were significantly reduced due to effective PEG shielding at neutral pH [43].

### 2.4. Poly(ε-caprolactone)-Block-Poly(ethylene glycol)-Small Molecular Ligand of Prostate Specific Membrane Antigen (PCL-PEG-SMLP) Synthesis and Biomedical Application Overview

**Figure 5:**
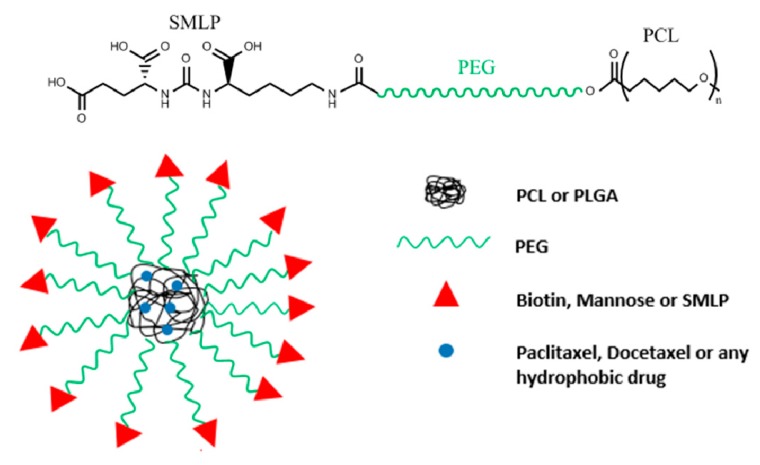
Chemical connectivity of PCL-PEG-SMLP nanoparticle design and nanoparticle design of those mentioned in Section 2.1, Section 2.3 and Section 2.4.

An approach for the synthesis of PCL-PEG-SMLP represented in Figure 5 would be starting with the copolymerization of PEG and PCL. OH-PEG-COOH added to ε-caprolactone (ε-CL) in the presence of a polymerization catalyst, stannous octoate, results in the copolymer PEG-PCL. The reaction proceeds via the ring-opening of ε-CL initiated by the hydroxyl group of PEG [44,45]. Furthermore, the carboxyl group of the PEG is activated with 1-Ethyl-3-(3-dimethylaminopropyl) carbodiimide (EDC) acting as the cross linker to get the intermediate product PCL-PEG-NHS in the presence of NHS. PCL-PEG-NHS is then added to a solution of SMLP (small molecular ligand of prostate specific membrane antigen) able to substitute the NHS group with the primary amine of the SMLP to form an amide linkage [19].

PCL micelles coated with PEG-SMLP are prepared by dialysis. The PCL self assembles to form particle sizes less than 60 nm. In the course of dialysis, a drug can be loaded into the assembled PCL polymers (Figure 5). Docetaxel (DTX)-loaded polymeric micelles DTX/PCL-PEG-SMLP targeting LNCaP cells for prostate cancer treatment were prepared.

PSMA a well-known trans membrane protein over expressed on prostate cancer epithelial cells [46,47], has been shown to have great potential for prostatic cancer (PCa) therapy. The critical role of SMLP conjugation is facilitating micelle uptake in those pathogenic cells. Jin et al. synthesized PCL-PEG-SMLP copolymers with different block lengths with optimized short-term stability (7 days) and drug-loading content. The targeted PCL-PEG-SMLP docetaxel-loaded polymeric micelles were able to deliver four different DTX-PMs to a prostate specific membrane antigen (PSMA) and positive prostate adenocarcinoma cells (LNCaP), exhibiting therefore a higher toxicity on LNCaP cells [19].

### 2.5. Gold Nanopartilces-PEG-TAT Peptide (AuNp-PEG-TAT Peptide) Synthesis and Biomedical Application Overview

**Figure 6:**
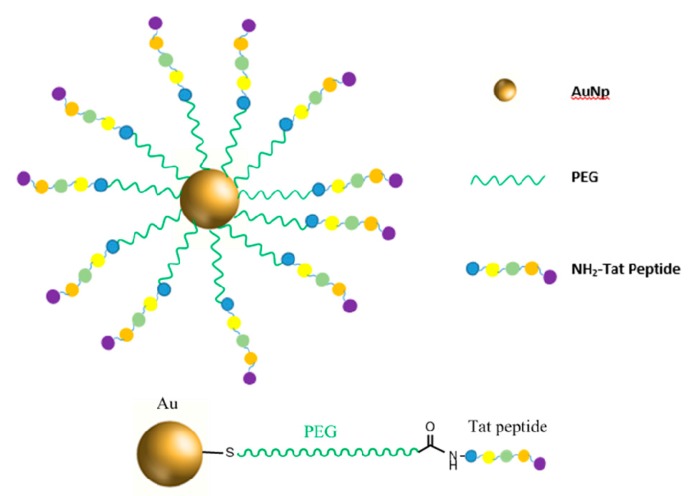
Targeted AuNp-PEG-Tat nanoparticle and chemical connectivity design.

Cell penetrating peptides (CPPs) have the ability to enter cells through endocytosis [48,49]. They are widely used in carriers to enable the entry of macromolecules attached to CPPs for delivery of various agents such as siRNA, nucleic acids, proteins, drugs, dyes, and fragments of DNA [50,51]. Very recently, Silva et al. published a review on the combination of CCPs with nanoparticles for therapeutic applications [52,53]. Among CPPs, Tat peptide demonstrated high efficiency to translocate small gold nanoparticles into the nucleus [54]. AuNp-PEG-*TAT peptide* can be synthesized starting with the attachment of thiol-PEG-carboxylic acid (SH-PEG-COOH) on gold nanoparticles via simple mixing. After attachment, the free end carboxylic groups of PEG were then mixed with Cpp derived from human immunodeficiency virus (HIV) Transcriptional Activator Protein (Tat), EDC, and Sulfo-NHS to activate the carboxylic group on the SH-PEG-COOH and then substitute it for an amine group found on the Tat via nucleophilic substitution. The AuNps-SH-PEG-TAT peptide (Figure 6) were incubated with Hela cells and found to be much more effective in killing the cells upon X-ray irradiation compared with unmodified AuNps and free TAT. This enhancement can be attributed to the better uptake of AuNps-SH-PEG-TAT into cancer cells. In addition, AuNps-SH-PEG-TAT was able to generate more oxygen reactive species to kill the cells as opposed to free TAT and AuNps [26]. Sanz et al. attached biofunctional thiolated poly (ethylene glycol) (PEG)-TAT onto 14 nm AuNPs-citrate, and demonstrated their effectiveness in oligonucleotides loading (dsRNA) by weak interaction (hydrogen bounds) between PEG chains spacers and dsRNA [55]. Very recently, Su et al. synthesized AuNPs-PEG-TAT with Iodine ^131^I that was labeled to AuNPs (^131^I-AuNPs), TAT (^131^I-TAT), and AuNPs-PEG-TAT (^131^I-AuNPs-TAT), respectively, for improved cell nucleus uptake and enhanced radiotherapy efficiency in colon cancer [56].

## 3. Synthesis of Modified Bio-Conjugated PEG Nanoparticles Acting as Tracking Agents

Traditional clinical imaging techniques such as computed X-ray tomography (CT), magnetic resonance imaging (MRI), and ultrasound (US) do not always meet the need of personalized cancer diagnosis because of the shift toward the development of precise diagnosis of diseases, particularly cancer [57]. Those methods are disadvantaged by impaired target specificity and inadequate information on the lesion location in the case of cancer [58,59,60]. Consequently, molecular imaging has received much attention recently. Molecular imaging denotes the formulation of molecular probes for the observation of cellular behavior, characterization, and the measurement of molecular processes in living organisms at the cellular and molecular level without interfering with them [61]. Therefore, scientists are interested in monitoring the distributive property of a prodrug, knowing the time a carrier spend in the body and confirming whether the carrier reached the target. This evidence is expected to have a major influence on tumor detection, individualized treatment, and drug advancement [62]. In this part, an overview of common nanocarrier-PEG-dye conjugates used for imaging is presented.

### 3.1. Coumarin-PEG-Au Synthesis and Biomedical Application Overview

The synthesis of coumarin-PEG-thiol can be achieved first by monotosylation of the PEG-diol using the method of Bouzide and Sauve (2002) [63]. Monotosylation is attributed to the added Ag_2_O that deforms the PEG molecule in a semi-circular fashion. The two hydroxyl groups are now close to each other causing internal hydrogen bonding between the two hydroxyl groups. The two hydroxyl groups now have slightly different acidities so the more acidic hydrogen can now be deprotonated by Ag_2_O. Now the oxygen is more nucleophilic for attacking the sulfur of the tosyl chloride (TsCl). The tosylate group (TsO^−^) obtained from TsCl of PEG-OTsis a much better leaving group than –OH. Potassium iodide acts as a catalyst by substituting for the chloride of the tosyl chloride. Iodine ion (I^−^) is a much better leaving group than Chloride (Cl^−^) thus facilitating the reaction. Many methods now can be used to replace the tosyl chloride with thiol group. One way is by adding the salt potassium thioacetate. Then a direct nucleophilic attack takes place replacing the the tosyl group with a –thioacetate group. To a solution of the monothioacetate-PEG-OH, coumarin isocyanate is added. The reactivity of isocyanates is attributed to the weak electron density at the carbon atom, therefore, isocyanate reactions primarily take place through addition to the C=N double bond. An active hydrogen atom-containing nucleophilic center attacks the electrophilic carbon atom and the active hydrogen is added to the nitrogen atom. The hydroxyl group of the OH-PEG-monothioacetate attacks the electron deficit carbon atom and a urethane group forms, holding the coumarin and PEG together [64,65,66,67]. The following reaction is the reduction of the thioacetate group to sulfur [68].

**Figure 7:**
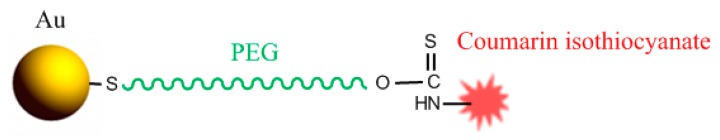
Chemical connectivity of Coumarin-PEG-Au nanoparticle design.

The thiol group can now be attached to the surface of gold nanoparticles acting as the carrier. Coumarin-PEG-Au nanoparticles (Figure 7) are used for intracellular tracking in human breast carcimoniaMDA-MB-231 cells. They can be tracked with nanometer accuracy. Within 1 h, the emission from bound dye was measured with a fluorescence spectrophotometer and these nanoparticles were rapidly internalized in the cells through non-specific endocytotic pathways and were found in the perinuclear region [20].

### 3.2. TAMRA-PEG-Au Synthesis and Biomedical Application Overview

**Figure 8:**
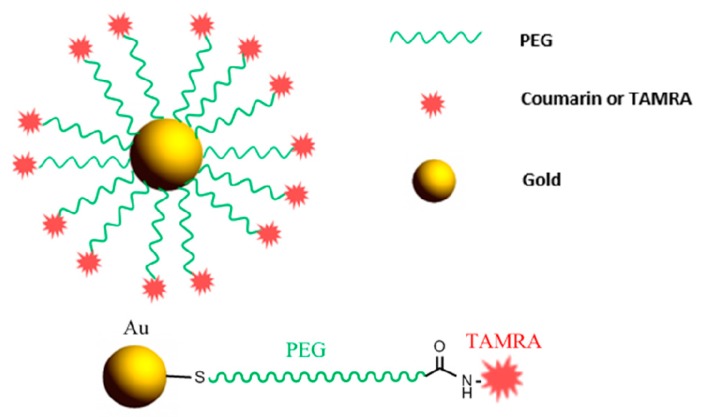
Targeted nanoparticle presented in Section 3.1 and Section 3.2 and chemical connectivity of TAMRA-PEG-Au nanoparticle design.

Heterobifunctional PEG (SH-PEG-COOH) is attached to gold nanoparticles in a basic media. Alkaline conditions facilitate deprotonation of the thiol terminal, which, in this way, attaches faster to the Au surface [69]. The carboxylic terminal of the PEG is conjugated to ((5(6)-carboxytetramethylrhodamine, TAMRA) using EDC conjugation chemistry [70,71]. Therefore, the -COOH group of the PEG is activated using EDC. Now an intermediate step can occur by adding NHS ester or directly adding the TAMRA to form an amide bond linkage between TAMRA and PEG [72] resulting in TAMRA-PEG-AuNP (Figure 8).

The TAMRA just serves as a tag for qualitative fluorescence imaging of the NPs that have been internalized by cells and therefore quenching does not mislead quantification experiments due to the proximity of Au nanoparticles [21].

### 3.3. Near Infrared Conjugated PEG Nanoparticles

Although most fluorophores function in visible or ultraviolet parts of the spectrum, near infrared (NIR) area can be very beneficial for fluorescence detection and imaging. Red emitting fluorescent dyes are suitable in life science applications such as antibody and protein labeling. In general, most biological tissues are transparent above 700 nm (the real value depends on tissue type) [73,74], thus providing much less background fluorescence from biological samples, reduced light scattering, high tissue penetration, and less sample damage than UV visible wavelengths. This allows for more convenient and detailed imaging of organisms [75,76,77].

### 3.4. Graphene Oxide GO-PEG-Cy7 Synthesis and Biomedical Application Overview

**Figure 9:**
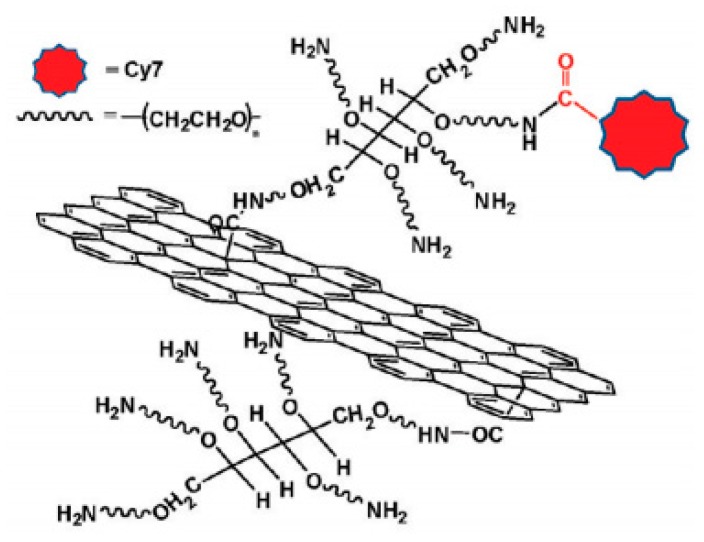
Graphene oxide (GO)-PEG-Cy7 nanoparticle design (obtained with permission from [22]).

The activation of graphene oxide (GO) surfaces occurs by nucleophilic substitution reaction of the hydroxyl groups on the grapheme oxide with chloroacetic acid in the presence of NaOH to convert the –OH groups to a more reactive –COOH group (GO-CH_2_-COOH) [78,79,80]. Then, six arm amine-PEG is conjugated with GO-CH_2_-COOH through the activation of the carboxylic acid moiety with EDC and the formation of an amide bond. The remaining amines of the PEG are used for further attachment such as an infrared labeling molecule Cy7 through amide bonds (Figure 9) [22].

In vivo fluorescence imaging discloses high tumor uptake of graphene oxide nanoparticles (NGS) in several xenograft tumor mouse models. PEGylated NGS show remarkable in vivo activities including highly efficient tumor passive targeting and quite low retention in reticuloendothelial systems, unlike PEGylated carbon nanotubes. The strong optical absorbance of NGS in the near-infrared (NIR) region was used for in vivo photothermal therapy in order to attain ultra-efficient tumor ablation after irradiation with low-power NIR laser on the tumor [22].

### 3.5. Poly(L-leucine)-Block-Poly(ethylene glycol)-Block-Poly(L-leucine)(PLL-PEG-PLL) Synthesis and Biomedical Application Overview

**Figure 10:**
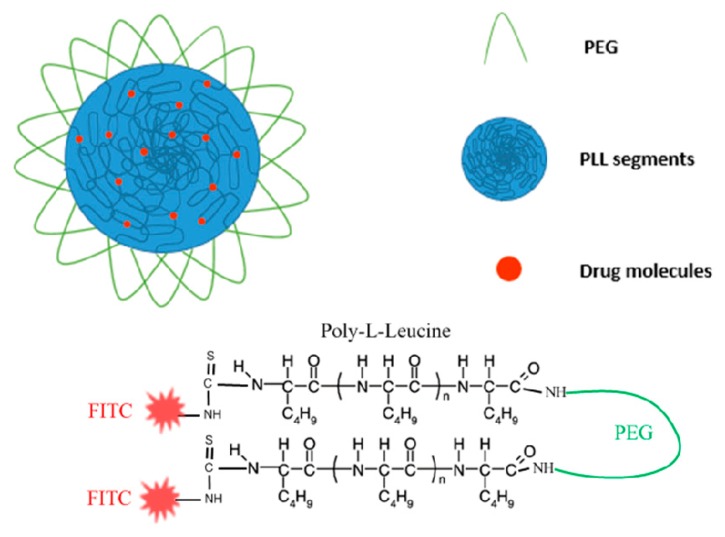
PLL-PEG-PLL chemical structure and nanoparticle design (nanomicelles) for drug loading and delivery.

Triphosgene, an in-situ supplier of phosgene is reacted with L-leucine to give a highly reactive cyclic monomer L-leucine N-carboxyanhydride (L-LeuNCA) [81]. Triphosgene reacts by nucleophilic attack on the carbonyl carbon by the primary nitrogen of the amino acid of L-leucine [82]. The highly reactive monomer then undertakes a ring opening mechanism (ROP) to polymerize L-leucine [83]. The initiator of the L-LeuNCA is an aliphatic primary amine Bis-AminePEG (BAPEG). The reaction follows the nucleophilic addition mechanism. The primary amine of BAPEG attacks the C5 carbonyl carbon of the L-LeuNCA to form an amide bond and the amino group of the L-Leucine becomes the initiator of the second attack and so forth [27,84]. After forming the copolymer PLL-PEG-PLL, the free end amines were fluorescently labeled by reacting with Fluorescein Isothiocyanate (FITC) (Figure 10). Micelles now can be formed via dialysis and entrapping a drug in the middle hydrophobic (poly L-Leucine) part of the micelles (Figure 10).

The micelles were internalized in Hela cells confirmed by the fluorescence of FITC attached to PLL. Prednisone Acetate and Paclitaxel loaded PLL-PEG-PLL micelles were used to assess the drug release of the micelles. Prednisone acetate-loaded PLL-PEG-PLL micelles had a slower diffusion rate than paclitaxel because it forms a tighter core with the PLL segments [27].

## 4. Synthesis of Modified Bio-Conjugated PEG Nanoparticles Acting as Drug Carriers

Small molecular drugs, especially the antitumor agents, often suffer problems such as low solubility, high toxicity, rapid excretion, or untargeted biodistribution [85]. To overcome the obstacles, one promising approach is to use a PEGylation strategy.

Compared to biological macromolecules, small organic molecules present fewer problems in the chemistry of PEGylation because they have fewer functional groups, lower conformational constraints, and easier purification and characterization steps [86,87].

Generally, permanent PEGylation requires low molecular-weight PEGs (Mw < 1000 Da) because macromolecular PEGs may block activity of small active agents at the target cells via steric hindrance. For releasable PEG attachments, namely the “prodrug approach,” the conjugate must be chemically or enzymatically transformed into their active form after administration [85,88,89,90]. Usually scientists prefer the entrapment of the drug in a hydrophobic core, to avoid the interactions of the drug with surrounding biomolecules, then upon a certain stimulus the drug is released.

### 4.1. Ibuprofen/PEG-Chitosan Synthesis and Biomedical Application Overview

Chitosan is a deacetylated polysaccharide, derived from chitin. Increasing the degree of deacetylation of chitosan occurs through the base hydrolysis of the amide in chitosan. The hydrolysis starts with the attraction of the present hydroxide ions to the carbon center of the acyl group of the amide [91,92]. The resulting primary amine is then protected with sodium dodecyl sulfate by mixing acidic solutions of chitosan and SDS (sodium dodecyl sulfate) [93]. The small amount of acid facilitates the solubility and protonation of the primary amine group for better electrostatic attraction with the negatively charged base dodecyl sulfate [23].

**Figure 11:**
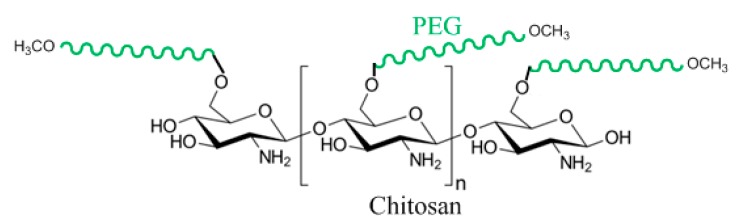
Chemical connectivity of Ibuprofen/PEG-Chitosan nanoparticle design.

Activated mPEG by NaH is reacted with chlorinated chitosan/SDS complex by oxalyl chloride as a chlorinating agent. Sodium hydride is a much stronger base than alcohol therefore the hydride ion is protonated (H_2_). The alkoxide OCH3-PEG-O^−^ acts as the nucleophile and substitutes the Cl^−^ of the chlorinated chitosan/SDS complex resulting in an ether connectivity [94]. The PEGylated chitosan (Figure 11) is then precipitated in Tris (hydroxyl methyl) amino methane aqueous solution to remove the SDS surfactant because SDS is soluble in the Tris aqueous solution at almost any concentration [93].

For preparation of ibuprofen encapsulated nanoparticles, a predetermined amount of drug was added to a PEGylated chitosan solution. Nanoparticles were formed after the drop wise addition of TPP (sodium Tripolyphosphate) of pH 3 to the PEGylated-chitosan solution of a pH of 5 containing the ibuprofen under constant magnetic stirring [95].

PEG-Chitosan/Ibuprofin nanoparticles were tested in vitro to evaluate their absorption in the colon. The results revealed more stabilized release of Ibuprofen from the PEG-Chitosan/Ibuprofin nanoparticles than that of unPEGylated chitosan [23].

### 4.2. Aspirin/Curcumin/mPEG-PLGA Synthesis and Biomedical Application Overview

After synthesizing mPEG-PLGA using a number of different approaches [96,97], using one of them previously mentioned, mPEG-PLGA nanoparticles were prepared via a modified o/w single-emulsion solvent evaporation process [98]. During their preparation, SH-Aspirin and/or Curcumin was dissolved with the mPEG-PLGA copolymer. The prepared SH-Aspirin/Curcumin-coloaded mPEG-PLGA nanomicelles had high drug-loading capacity and stability. The authors observed obvious synergistic anticancer effects on ES-2 and SKOV3 human ovarian carcinoma cells in vitro, and activation of the mitochondrial apoptosis pathway was shown [24].

### 4.3. Ridaforolimus/NH_2_-PEG-DSPE Synthesis and Biomedical Application Overview

**Figure 12:**
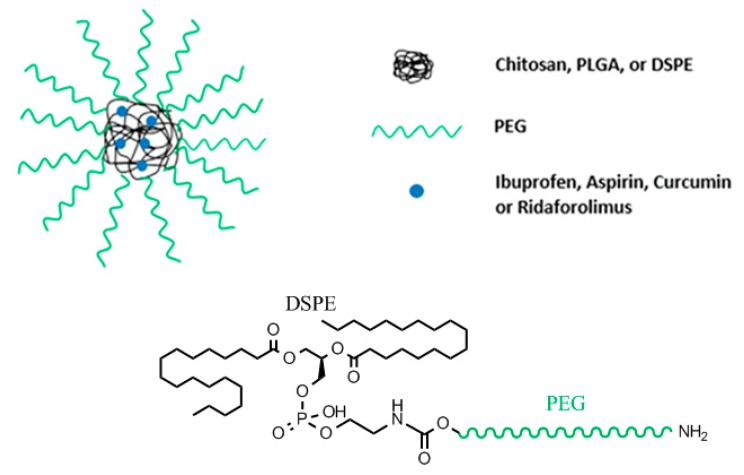
Nanoparticle design of those presented in Section 4.1, Section 4.2 and Section 4.3, and chemical connectivity of Ridaforolimus/NH_2_-PEG-DSPE nanoparticle design.

Beginning with NH_2_-Peg-OH, the amino group of the heterobifunctional PEG was selectively protected by the butyloxycarbonyl group (Boc). Then, a succinimidyl carbonate (SC) group was introduced at the hydroxyl end of Boc-PEG-OH by substitution reaction, to later form a urethane linkage between the amino group of 1,2-Distearoyl-sn-glycero-3-phosphoethanolamine (DSPE) and the introduced succinimidyl carbonate group. The primary amine functionality of the PEG gets regenerated by the acidolytic removal of the Boc group (Figure 12) [99,100].

Micelles of DSPE-PEG encapsulating a drug (Figure 12) were prepared by solvent evaporation technique. The micelles were loaded with the drug Ridaforolimus. Ridaforolimus has been shown to inhibit mTOR, a serine/threonine kinase protein [101,102,103]. Its inadequate activation could lead to a number of cancers and could be responsible for tumor growth and multiplicity. Ridaforolimus’s high lipophilicity requires either a change in structure of the drug or an appropriate macromolecule to carry the drug [102]. It was found that loading Ridaforolimus into DSPE-PEG nanomicelles greatly improved the solubility of Ridaforolimus by approximately 40 times. Additionally, the micelles improved retention of the drug in the plasma by increasing the half-life of the drug by 170% and decreasing its clearance by 58% [101,104,105].

### 4.4. Doxorubicin/MSN-Gelatin-PEG Synthesis and Biomedical Application Overview

*N*-Cetyltrimethylammonium bromide (CTAB) is a surfactant added to a basic aqueous solution of NaOH to act as template, which will contribute to the pores of the mesoporous (MSN) silica. Tetraethylorthosilicate (TEOS) and (3-aminopropyl) triethoxysilane (APTES) were then added to start forming the silica network. These two chemicals are responsible for the silica network starting with their hydrolysis and then polycondensation. APTES provides the amine functionality on the pores due to the 3-aminopropyl directly connected to the silica, which is not involved in the mentioned reactions. Once the gel is formed, it is washed to remove byproducts of the reactions and after that, the CTAB is calcinated at high temperatures forming the network [106]. The benefit of having amine functionality was to insert a targeting moiety for the particles.

**Figure 13:**
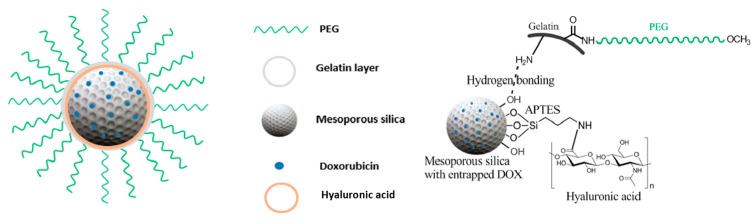
Doxorubicin/mesoporous silica nanoparticles (MSN)-Gelatin-PEG nanoparticulate design.

The nanoparticles were loaded with the drug Doxorubicin (DOX) simply by mixing the mesoporous silica nanoparticles (MSN) with a solution of DOX and stirring them. The drug gets entrapped in the porous network of the MSN (Figure 13) [25]. After loading the MSN particles with DOX, the particles were partially blocked with a tumor-targeting ligand hyaluronic acid through the amidation reaction between the –COOH group of HA and amine groups on silica particles. After capping with HA, the particles are coated with gelatin. The interaction occurs through hydrogen bond interaction between amine groups available in the gelatin and the hydroxyl groups on the MSN particles [107]. The gelatin (GEL) is then cross-linked by glutaraldehyde (GA) by formation of bonds between amine groups in GEL and GA [108]. Gelatin also expresses carboxylic groups which will be activated using EDC to further attach mPEG-amine to the particles [107]. In vitro results pointed out that this system attained improved cellular uptake performance and astonishing killing effectiveness on CD44-positive MDA-MB-231 cells [25].

### 4.5. Metal Organic Frameworks (MOFs) Synthesis and Biomedical Application Overview

Metal organic frameworks (MOFs) are polymer metals composites consisting of metal ions or clusters coordination sites linked together by organic functional groups (ligands) usually synthesized by solvothermal reactions [109]. MOFs can have diverse crystalline nanostructures of very high porosity due to permanent voids, allowing high loading capacity of biomolecules and making them very attractive vehicles for drug delivery [110,111]. Furthermore, MOFs surfaces can be easily modified with biomolecules that enhance their biocompatibility [112]. For example, D. Wang et al., used a layer-by-layer method to synthesize a core-shell MOF (MIL-100(Fe)) coated photothermal agent Prussian blue nanocubes PB@MIL-100(Fe) with dual metal-organic-frameworks (d-MOFs). The d-MOFs served as a T1-T2 dual-modal magnetic resonance imaging (MRI) contrast and fluorescence optical imaging (FOI) agent, due to the existence of inner PB MOFs and outer MIL-100(Fe) MOFs. The authors tested d-MOFs combined theranostic effects both in vitro and in vivo, and demonstrated that artemisinin (a traditional Chinese anticancer medicine) with a high loading content of 848.4 mg/g is released from the d-MOFs upon tumor cellular endocytosis due to the pH-responsive degradation of outer MOFs in low pH lysosomes of tumor cells, while the inner PB MOFs was used for photothermal therapy due to its strong absorbance in the NIR region [113]. Very recently Guangxu Lan et al. published a review paper on the nanoscale metal–organic frameworks for phototherapy of cancer [114], while Qun Guan et al. and Kuangda Lu et al. reported on their Photodynamic Therapy use in cancer treatment and their application in sensing and imaging respectively [115,116]. Herein, we will discuss a few examples found in the literature on PEGylated-MOF nanostructures and their use in nanotheranostics. Aba’ nades La’ zaro et al. synthesized 200 nm nanoparticles zirconium terephthalate MOF UiO-66 with covalently PEG modified surface using the click modulation protocol [117]. The authors demonstrated the importance of PEG coating in enhancing the stability of MOF toward phosphates at pH 7.4 as well as ‘‘burst release’’ phenomenon by blocking interaction with the exterior of the nanoparticles, while at pH 5.5 PEG allowed a stimuli-responsive drug release. Moreover, their results show that a PEGylated UiO-66 nanovector potentially escapes lysosomal degradation through enhanced caveolae-mediated uptake. Zang et al. reported, for the first time, a one pot process for the synthesis of a biocompatible zeolitic imidazolate framework ZIF-8 nanovehicle with high drug loading and a pH-triggered release behavior for co-delivery of verapamil hydrochloride (VER) as a P-glycoprotein inhibitor and doxorubicin hydrochloride (DOX) as an anticancer drug. The authors coated the nanoparticles with methoxy poly (ethylene glycol)-folate (PEG-FA) and demonstrated that coating ZIF-8 with PEG-FA can enhance the stability of (DOX+VER)@ZIF-8 by reserving the multidrug resistance and achieving prolonged circulations as well as an active targeting drug delivery detected by near infrared fluorescent (NIRF) imaging, and resulting in high therapeutic efficiency both in vitro and in vivo [118]. Similarly, Shi Z et al. synthesized a multifunctional drug delivery system (DDS) based on a targeted methoxy poly (ethylene glycol)-folate (FA-PEG) coated zeolitic imidazolate framework (ZIF-8) with high loading capacity that disintegrates at low pH resulting in an effective targeting delivery and release of chloroquine diphosphate (CQ) as an autophagy inhibitor into HeLa cells [119]. Giménez-Marqués et al. reported a the highly selective and general grafting GraftFast method for successful attachment of multifunctional biopolymers (polyethylene glycol (PEG) and hyaluronic acid) on the external surface of iron trimesate MIL-100(Fe) nanoparticles (NPs) leading to suitable chemical and colloidal stability in different biofluids, with a conserved porosity and adsorption efficiency of bioactive molecules [120]. Furthermore, using high-resolution soft X-ray spectroscopy the authors demonstrated that the radio-labeled antitumor agent gemcitabine monophosphate (3H-GMP)-loaded MIL-100(Fe)@PEG NPs shows reduced macrophage phagocytosis, confirming a significant in vitro PEG furtiveness.

### 4.6. DOX/MCN-PEG Synthesis and Biomedical Application Overview

**Figure 14:**
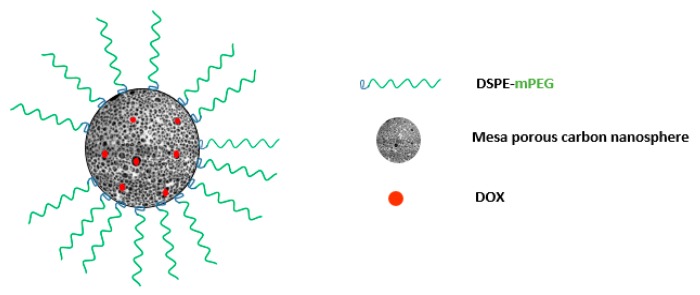
Doxorubicin hydrochloride (DOX)/MCN-PEG nanoparticulate design.

The carbonaceous structure of Mesoporous Carbon Nanospheres (MCNs) is usually very hydrophobic and needs to become hydrophilic to enable surface modification and use in the body. A common approach is the oxidation of MCNs using a strong acid (e.g., H_2_SO_4_ and HNO_3_) [121,122,123]. This will create a number of oxygen functional groups on the surface of MCNs. MCNs are usually synthesized by calcination or hydrothermal treatment [124,125]. Spherical phenolic resol mixed with Pluronic F127 (acting as a template), forms monomicelles through hydrogen bonding between the resol and Pluronic F127. After increasing the temperature to crosslink the resol/F127 monomicelles to form a spherical cluster of resol/F127 combination, the template is removed by increasing the temperature to about 700 °C under flow of nitrogen [124]. Carboxyl and hydroxyl groups are introduced after mixing with concentrated nitric and sulfuric acid. DOX loading was carried out by simply mixing DOX solution with oMCNs (oxidized MCNs) in a phosphate-buffered saline (PBS) at pH 9.0. Then, the addition of DSPE-mPEG is adsorbed onto the surface of oMCNs by means of hydrophobic-hydrophobic interaction between DSPE and the oMCNs (Figure 14) [126,127]. DSPE-mPEG is introduced to prevent the recognition of the reticuloendothelial system [128,129]. Only oMCN/DOX/PEG exhibits some increase in the concentrations of DOX in plasma compared with those of free DOX or oMCN/DOX. This is because free DOX and oMCN/DOX might be easily identified by the reticuloendothelial system and rapidly cleared from plasma without the protection of PEG [121].

## 5. Conclusions

Various nanoparticulate systems can incorporate PEG coatings to shield their surface from aggregation, opsonization, phagocytosis, and prolonging systemic circulation time. The success of PEG is also attributed to its easy chemical modification to conjugate to almost every nanoparticle setup. As a result, nowadays PEG is the most used polymer in the biomedical field of drug delivery, targeting, and imaging, and the only polymeric therapeutic that is market approved for different drugs. Multimodal macromolecules usually incorporate the three parts of the review discussed here, which means formulating a nanosystem with three or more functions to target, deliver, and track simultaneously.

Nevertheless, many of the synthesized nanoparticles lack extensive pharmacokinetic and pharmacodynamics studies. Moreover, research obtained in recent years show that PEG may have possible drawbacks, such as interaction with the immune system, probable degradation under stress, and accumulation in the body if not excreted properly. Additional investigation on pharmacokinetic and pharmacodynamics of both the assembled nanosystems and PEG individually would greatly help develop more successful nanoparticles with more uniformity, drug loading, and release ability.

Further areas of research may include finding an alternative polymer to PEG, or making broad different synthetic polymers accessible, some of which are poly(glycerol)s, poly (amino acid)s, poly-(vinylpyrrolidone), poly (2-oxazoline)s, and poly (N-(2-hydroxypropyl) Methacrylamide. However, Poly (amino acid)s are the only biodegradable polymers and provide a stealth effect. Therefore, it is always of interest to shift the research toward finding the “ideal” polymer with the fewest side effects to complement nanocarriers and make a safe “in and out” journey through the body.

## Figures and Tables

**Table 1:** The nanostructures presented in this review and their preparation methods.

Nanoparticle System	Pathways for Their Synthesis	Biomedical Applications
PCL-PEG-Biotin [16]	Activation of Biotin. Addition of PEG bis amine. Polymerization with ε-caprolactone.	Targeted paclitaxel chemotherapy drug to MCF-7 and HeLa cells.
Magnetite-PEG-Folate [17]	Coprecipitation of the two salts FeCl_3_·6H_2_O and FeCl_2_·6H_2_O. Addition of oleic acid and oleylamine for stabilization. Addition of PEG. Addition of activated folic acid.	Targeted delivery of doxorubicin (DOX) to HeLa cells.
PLGA-PEG-Mannose [18]	PEGylation of mannose by Mono t-BOC-protected PEG bis amine. Removal of t-BOC group. Reaction with activated PLGA.	Delivery of amphotericin B to macrophages via enhanced macrophage targeting and mannose-mannose uptake.
PCL-PEG-SMLP [19]	Copolymerization of PEG and PCL. Activation of PCL-PEG-COOH with EDC and NHS. Reaction with SMLP.	Specific delivery of four different (DTX-PMs) to a (PSMA) positive prostate LNCaP cells.
Coumarin-PEG-Gold [20]	Reaction of monothioacetate-PEG-OH with Coumarin isocyanate. Reduction of monothioacetate-PEG-Coumarin to SH-PEG-Coumarin. Attachment to Gold nanoparticles.	Rapid internalization and intracellular tracking in MDA-MB-231 cells.
TAMRA-PEG-Gold [21]	Attachment of SH-PEG-COOH to gold nanoparticles. Activation of Au-PEG-COOH with EDC. Reaction with TAMRA.	Qualitative fluorescence imaging of the internalized AuNPs.
GO-PEG-CY7 [22]	Activation of graphene oxide with chloroacetic acid. Activation of –COOH groups on GO-CH_2_-COOH with EDC. Conjugation of six-arm branched amine PEG on GO. Addition of Cy7 infrared dye.	In vivo fluorescence imaging in xenograft tumor mouse models.
Ibuprofen/Chitosan-PEG [23]	Increasing the degree of deacetylation of chitosan. Protection of the amine of chitosan with SDS. Reaction of NaH activated PEG with chlorinated Chitosan/SDS complex. Removal of the SDS surfactant. Addition of Ibuprofen drug and using TPP solution to form nanomicelles.	Encapsulation of ibuprofen, a poor water soluble drug, and in vitro release in gastrointestinal and simulated biological fluids.
Aspirin/Curcumin/PLGA-mPEG [24]	Preparation of PLGA-mPEG copolymer. Adding a predetermined amount of SH-Aspirin and/or Curcumin. Nanoparticles formation using a modified single-emulsion solvent evaporation process.	Synergistic anticancer effects on ES-2 and SKOV3 human ovarian carcinoma cells in vitro, and activation of the mitochondrial apoptosis pathway.
Doxorubicin/MSN-Gelatin-PEG [25]	Addition of CTAB surfactant. Hydrolysis and polycondensation of TEOS and APTES. Calcination of CTAB surfactant. Mixing Doxorubicin with the MSNs. Capping with hyaluronic acid. Coating, crosslinking and activating Gelatin with EDC respectively. Conjugation of mPEG-amine to the Gelatin.	In vitro improved cellular uptake and astonishing killing effectiveness to CD44-positive MDA-MB-231 cells.
AuNP-PEG-*TAT Peptide* [26]	Attaching SH-PEG-COOH on AuNps. Activation of –COOG group with EDC and NHS. Substitution of activated –COOH group with Tat peptide.	Enhanced cellular uptake by Hela cells in vitro, and effectiveness in k generation of more oxygen reactive species resulting in cell death upon X-ray irradiation.
PLL-PEG-PLL [27]	Reaction of Triphosgene with L-Leucine. Polymerization of L-LeuNCA using BAPEG as initiator.	Drug loading and in vitro drug release.
